# Predicting Human Aluminium Exposure from Vaccinations Using a Physiologically-Based Toxicokinetic Model

**DOI:** 10.3390/vaccines14040346

**Published:** 2026-04-14

**Authors:** Karin Weisser, Niklas Hartung, Gaby Wangorsch, Wilhelm Huisinga, Brigitte Keller-Stanislawski

**Affiliations:** 1 Division Safety of Biomedicines and Diagnostics, Paul-Ehrlich-Institut (Federal Institute for Vaccines and Biomedicines), Paul-Ehrlich-Straße 7, 63225 Langen, Germany; gaby.wangorsch@pei.de; 2 Institute of Mathematics, University of Potsdam, 14476 Potsdam, Germany; niklas.hartung@uni-potsdam.de (N.H.); huisinga@uni-potsdam.de (W.H.); 3 Paul-Ehrlich-Institut (Federal Institute for Vaccines and Biomedicines), Paul-Ehrlich-Straße 7, 63225 Langen, Germany

**Keywords:** aluminium, adjuvants, toxicokinetics, vaccination, computer simulation

## Abstract

**Background/Objectives**: Poorly soluble aluminium (Al) compounds have successfully been used for decades as adjuvants in vaccines, enabling an effective immune response. Yet the safety of Al exposure from vaccines is consistently questioned, especially regarding infants. Since toxicokinetic data of aluminium after vaccination in humans are not available, model-informed predictions are needed for risk assessment. **Methods**: Using a physiologically-based toxicokinetic model, we predicted the Al exposure from i.m. injections of Al-adjuvanted vaccines for full-term neonates to 50-year-old adults following the recommended vaccination schedule in Germany 2025 in addition to the continuous oral background Al exposure from dietary intake. **Results**: During the first two years of life, moderate (max. 2-to-3-fold) but transient increases of Al concentrations in plasma and in the relevant target organs liver and bone due to vaccinations were predicted. Increase in brain Al content was 4%. Most importantly, in all tissues, maximum Al levels did not exceed normal levels observed in infants soon after birth or known from adults. In children and adults, the rise in Al concentrations in plasma and tissues due to single vaccinations was marginal. The calculated contribution of vaccinations to the Al body burden at age 50 was negligible. **Conclusions**: From a toxicokinetic perspective, the additional Al exposure in full-term infants, children and adults from vaccinations with Al-adjuvanted vaccines according to the current recommended schedules is considered safe. The model has proven a valuable tool for predictions of Al exposure from vaccinations.

## 1. Introduction

Many important vaccines are adsorbed on aluminium (Al) adjuvants, e.g., the toxoid vaccines against diphtheria and tetanus, acellular pertussis, hepatitis B, and pneumococcal and meningococcal vaccines. Al adjuvants potentiate the immune response to the poorly immunogenic antigens and thereby enable a successful vaccination. In Europe, the content of Al in vaccines is limited by the European Pharmacopoeia to a maximum of 1.25 mg Al per dose [1].

Al compounds used as vaccine adjuvants mainly consist of complex polymers of crystalline aluminium oxyhydroxide or amorphous aluminium hydroxyphosphate [2], referred to below for the ease of reading as AH (Al hydroxide), AP (Al phosphate) and AAHS (amorphous Al hydroxyphosphate sulfate). Vaccine antigens are typically adsorbed onto the surface of preformed adjuvants [3]. After vaccination the amount of injected Al can be considered as 100% systemically available, but due to poor solubility, intramuscular absorption is very slow. Both amorphous structure and higher solubility might contribute to the higher rate of systemic availability of AP compared to AH. Estimates from injection site measurements suggest that complete absorption takes about 3 months (AP) up to 1 year (AH) [4].

Al-containing vaccines have been used successfully and safely over decades [5,6]. The existing evidence has been supplemented recently by an epidemiological study from Denmark [7] that did not find evidence supporting an increased risk for autoimmune, atopic or allergic, or neurodevelopmental disorders associated with early childhood exposure to aluminium-adsorbed vaccines. Nevertheless, persistent concerns have been raised considering the fact that neonates and infants receive the same (or even higher) absolute Al amounts per vaccination than adults during the first year of life, while their kidney function, which is essential for elimination of Al from the body, is immature.

The concerns are compounded by the fact that the development of new vaccines and adapted recommendations over 40 years has led to a steady increase in Al exposure from vaccinations during the first two years of life. For example, the cumulative Al exposure via vaccination has increased 3 to 4 times in Germany since 1980, depending on the chosen brand.

Attempts to assess the risk based on comparison of cumulative amounts of Al exposure from various sources (e.g., by [8]) are limited because they do not account for (i) the rates of absorption from various routes of exposure (apart from the extent of absorption as 0.1–0.3% from diet vs. 100% from vaccines); (ii) the kinetic differences between a continuous intake of low amounts (as exposure from diet) and a singular administration of a high dose (as with vaccination), even if cumulative amounts are equal; (iii) the age-dependent physiology influencing Al kinetics; and (iv) the variable impact of an increase in (e.g., cumulative) exposure depending on the Al load reached in relevant target organs.

Physiologically-based toxicokinetic modelling allows us to overcome these difficulties by extrapolating from diverse kinetic data to specific predictions of exposures, esp. in children and infants. Moreover, a model-informed assessment can continuously be adapted, e.g., when Al content and vaccination schedules are subject to change.

In recent years we have closed the scientific gap identified in 2007, i.e., the absence of a physiologically-based toxicokinetic (PBTK) model being capable of predicting both plasma and tissue concentrations with sufficient accuracy [9]. We have developed a PBTK model for aluminium exposure in humans that is built on a comprehensive collection of animal and human toxicokinetic data [4,10]. The model was successfully validated by external data sets from animals and humans after s.c. and i.m. absorption from adjuvanted vaccines or immunotherapeutics [4]. It was recently applied to simulate Al exposure from subcutaneous immunotherapy (SCIT) in different age groups [11].

Herein we leverage the PBTK model to predict the joint Al exposure from diet and vaccinations from birth onwards comparing Al concentration time courses as well as the accumulated Al body burden over a lifetime. The predictions are intended to provide support for regulatory risk assessment and a science-based contribution to the discussions on the safety of Al in vaccines.

## 2. Materials and Methods

### 2.1. Simulation Scenarios

#### 2.1.1. Al Exposure from Dietary Intake

As described and used recently [4,11], a continuous age-dependent dietary intake scenario was simulated to generate background exposure levels of Al expected in healthy female subjects from birth to adulthood (50 years). In brief, based on results of European diet studies [12], anoral exposure of 0.8 mg Al/kg/week from food and water was assumed for children and adults (>12 months), and of 0.1, 0.2, 0.4 and 0.8 mg/kg/week for infants aged 0–3, 4–6, 7–9 and 10–12 months, resulting from infant formulae and other foods manufactured especially for infants. Further assumptions include an average oral bioavailability of 0.17% [4], and initial Al levels at birth built up in all organs during embryonic development (see Section 2.2).

This dietary intake scenario was used as the basis for simulations of additional Al exposure from vaccinations. As Al in food is the main source of dietary exposure, the term “food only” is used in the following.

#### 2.1.2. Al Exposure from Food and Vaccinations from Birth to Adulthood (0–50 Years)

Al exposure from vaccinations was simulated according to the vaccination schedule recommended for the whole population between 0 and 50 years of age in Germany 2025 ([13]; Table 1, white area). Vaccinations for special risk groups (known as indicated vaccinations) or for traveling purposes were not considered. Only vaccinations that contain Al adjuvants were included, thus not included are recommended vaccinations with live attenuated vaccines (e.g., measles-containing vaccines) or others not containing Al (e.g., inactivated influenza vaccines). For vaccination against human papillomavirus (HPV), the recommended 2-dose scheme was applied. All vaccinations were administered at the earliest age in the recommended period. In the case of more than one available product for a particular vaccination on the market in Germany 2025, the one with the higher Al content was used. Adjuvant type (AH: aluminium hydroxide; AP: aluminium phosphate; AAHS: amorphous aluminium hydroxyphosphate sulfate) and Al amount per vaccine dose of the products were retrieved from Section 2 of the summary of product characteristics (SmPC). Al exposure from vaccinations was predicted in addition to the background exposure from food (“food + vacc”; see Section 2.1.1).

#### 2.1.3. Al Exposure from Food and Vaccinations Including “Additional HepB at Birth”

Since vaccines against hepatitis B (HepB) are also Al-adjuvanted, and in some EEA (European Economic Area) countries [14], there is a general recommendation for HepB vaccination at birth, we added a second scenario with two additional HepB vaccinations at birth and 1 month (see Table 1, grey area). In Germany, as in most European countries, this schedule is only recommended for newborns of HepB-infected mothers. Two monovalent HepB vaccines for children using either AH or AAHS are available. For the predictions, we assumed an AAHS vaccine, due to the faster absorption rate, as the most conservative approach.

#### 2.1.4. Body Burden of Al from Food and Vaccinations After 50 Years of Life

In order to investigate the contribution of Al from vaccinations to the Al body burden at later ages, we calculated the total amount of Al present in the body of a reference female adult at the age of 50 years for both scenarios (food-only and food + vacc including “additional HepB at birth” from 0–50 years).

### 2.2. PBTK Model Structure and Simulation Details

Simulations were based on the PBTK model described in Hartung et al. 2025 [4]. This model is characterised by an age-dependent physiological parametrisation for full-term newborns to adults, combined with a model for GFR (glomerular filtration rate) maturation, and a unique bone submodel. In this submodel, uptake of Al as a bone-seeking element is assumed to be proportional to calcium (Ca) uptake, and Al release from bone identical to that of Ca. The Ca kinetic submodel captured these age-dependent bone remodelling processes from birth to adulthood and has been validated against diverse Ca kinetic data from the literature [15]. Brain is conservatively modelled as a sink due to the available experimental data base that shows no sign of release from the tissue [10]. The PBTK model has been successfully validated against a compilation of diverse literature data (mainly ^27^Al) for different age groups, routes of administration, and tissues for both short-term and long-term exposure [4].

In contrast to the recent simulations for SCIT [11], a particular focus here is on the suitability of the model for predictions in newborns and infants shortly after birth. Apart from the inclusion of two age-dependent physiological processes most relevant for Al kinetics, maturation of renal function and bone remodelling (see above), the model also accounts for the impact of the fetal Al exposure during pregnancy by assuming initial Al levels in all organs at birth. Organ-specific initial level distributions have previously been fitted to literature data from multiple sources [4]. In order to be fully in line with the plasma level predicted in our female adult after food-only exposure, ensuring quasi steady state conditions at birth, we had to slightly reduce the median initial level in plasma from 5.5 to 2.2 µg/L. This is at the lower end of the range of plasma levels of newborns reported in literature (2–15 µg/L; [4]). Compared to higher initial levels, this is considered a more conservative approach, since relative increases due to vaccination are more pronounced.

Inter-individual variability is implemented on model parameters (oral bioavailability, GFR and tissue distribution; [4]). The model further contains dosing modules for oral and parenteral routes of administration. For i.m. absorption of Al from adjuvants, a specific zero-order absorption rate was used for each adjuvant type (AH: 0.002784/day, AP: 0.01098/day) estimated from injection site release data after adjuvant administration in various animal species (rat, rabbit, monkey; see [4]). Since AAHS can be considered a form of AP adjuvant with similar physicochemical and dissolution properties [3], the same rate was applied as for AP. The i.m. absorption rates were assumed to be lognormally distributed in the population, with a 50% coefficient of variation [4].

Simulation results and statistical analyses were obtained using the software R, version 4.2.2 [16], specifically using the R package mlxR version 4.2.0 [17] for Al exposure predictions in tissues and plasma. To solve the system of ordinary differential equations (ODEs), we used the Monolix stiff ODE solver with adaptive step size and standard tolerances (absolute tolerance: 10^−9^, relative tolerance: 10^−6^). For graphical representation, weekly readouts were used.

For each simulation, we used an identical virtual population of N = 500 female individuals to ensure comparability amongst the scenarios. Predictions for both sexes confirmed that female subjects can be considered the toxicologically more sensitive population (see Figure S1 in [11]).

### 2.3. Data Retrieval and Evaluation

The predicted Al concentration–time curves after food + vacc exposure are displayed as median with lower (5% quantile, p5) and upper (95% quantile, p95) bounds. We graphically superimposed the food + vacc exposure levels on the median exposure expected from food only (dotted line) to highlight the increase in Al exposure attributed to vaccinations. Al concentrations (median, p95) in plasma and tissues were calculated at the timepoint of the highest median difference during infancy (0–2 years), between 9–10 years (after first HPV and TdaP-IPV booster vaccination) and 19–20 years (after TdaP booster vaccination). For all concentrations the differences and ratios of the corresponding medians after food + vacc compared to food only were calculated.

The toxicological evaluation of predicted total Al levels in tissues was based on data of upper limits of normal (ULN) or critical levels of Al in tissues reported in the literature. As done in [4,11], values published as µg/g dry weight (dw) were converted into Al amounts per wet weight (ww) to allow for a meaningful comparison with predicted Al tissue levels modelled as ww. For example, the conversion factor for bone samples (0.497) accounts for the difference between Al in dry bone samples and predicted Al concentrations in wet model bone tissue defined as cartilage-free and bone marrow-including (for details see [4] (chapter 2.2.3)).

A level of 5 µg/g ww was considered as ULN for Al content in bone of healthy adults (converted from the ULN of 10 µg/g dw reviewed by [18]). Levels > 30 µg/g ww are reported to be clearly associated with Al-induced bone disease and osteomalacia [18,19,20]. The lowest Al content in bone associated with osteomalacia symptoms (7 µg/g ww, herein referred to as “critical level”; converted from 14 µg/g dw) was found in one adult patient on long-term total parenteral nutrition (TPN), which constitutes a relevant source of Al due to contaminated PN solutions [21].

A derivation of a ULN is more uncertain for brain tissue. A range of 0.02–1 µg/g ww for ”normal” brains over several decades is reported (converted from 0.1–4.5 µg/g dw [22]); conversion factor 0.23; see [4]) with a clear trend of an increase with age [23]. Given the available data, we herein refer to 1 µg/g ww as a ULN for Al in brain of adults. A critical level of Al content in brain with respect to neurotoxicity is unknown.

A ULN of 4 µg/g ww in liver for adults was deduced from the range of 0.07–4.33 µg/g ww measured in 140 adult autopsy liver tissues in Germany (age range 21–81 years, [23]). Measurements in children showing hepatotoxic symptoms after TPN (18 to 34 months of age) were in the range of 8–40.5 µg Al/g ww (converted from 32–162 µg/g dw, [24]; conversion factor 0.25; see [4]), but the hepatic pathology observed was not clearly related to Al exposure from TPN only. Therefore, critical levels for the Al content in liver also cannot be inferred.

All ULNs, as well as toxicity-associated levels derived, are displayed in the figures as horizontal dashed lines.

Available data on tissue Al levels in healthy newborns and infants had been used to estimate initial levels at birth ([4]; see Section 2.2).

## 3. Results

### 3.1. Al Exposure from Food and Vaccinations from Birth to Adulthood (0–50 Years)

**Figure 1:**
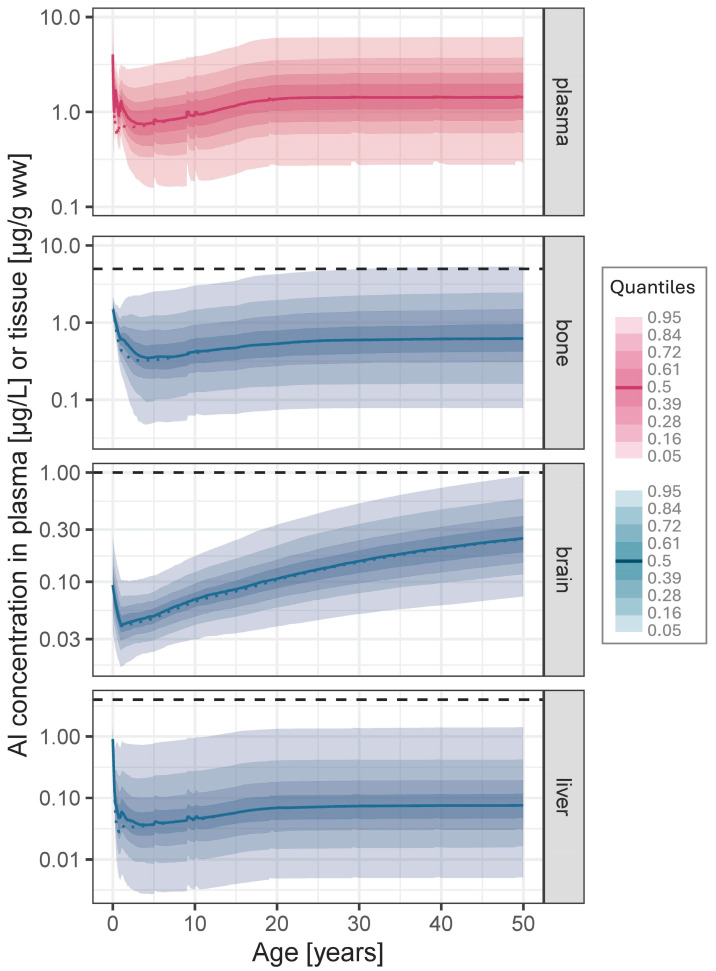
Predicted time courses of Al concentration in plasma, bone, brain and liver following joint exposure from vaccinations (according to the generally recommended vaccination schedule in Germany 2025 [12]; see Table 1) and continuous background dietary intake (food + vacc) from birth to the age of 50 (solid line: Median; coloured shaded areas: Quantiles; horizontal dashed line: Upper limit of normal (see Section 2.3); dotted line: Median time course of “food-only” exposure).

Figure 1 shows the predicted time courses of Al concentration in plasma and tissues from birth to 50 years of age following joint exposure from vaccinations recommended for the general population in Germany 2025 and dietary intake (food + vacc) compared to the median of the background dietary intake only (food only).

**Figure 2:**
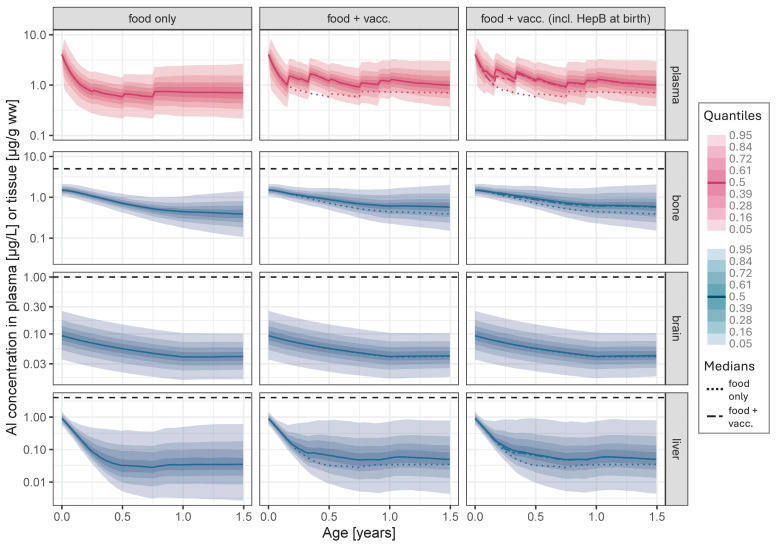
Predicted time courses of Al concentration in plasma, bone, brain and liver from birth to the age of 1.5 years following continuous background dietary intake (“food only”, left), joint exposure from vaccinations according to the recommended vaccination schedule in Germany 2025 (“food + vacc”, middle), and joint exposure from the vaccination schedule including two additional HepB vaccinations after birth (“food + vacc incl. HepB at birth”, right; adjuvant type: AAHS, see Table 1) (solid line: Median; coloured shaded areas: Quantiles; horizontal dashed line: Upper limit of normal (see Section 2.3); dotted line: Median time course of “food only” exposure; broken line in the diagrams on the right: Median time course of “food + vacc” exposure).

The main increase in Al levels in plasma and tissues due to vaccinations occurs during the first two years of life, with a peak shortly after the vaccinations at 4 months of age (see excerpt of years 0–1.5 in Figure 2, middle). Median Cmax in plasma reaches 1.6 µg/L (p95: 3.4 µg/L) corresponding to a 3.4-fold increase compared to food-only exposure (0.5 (1.5) µg/L; median difference: 1.1 µg/L; Table 2). A similar increase (2.6-fold; median difference: 0.04 µg/g ww) is observed in liver. Increase in bone is less pronounced (1.6-fold; 0.2 µg/g ww) and very small in brain (1.03-fold; 0.002 µg/g ww). Despite these increases, in all tissues, maximum Al levels during the first 2 years of life did neither exceed the initial levels at birth nor the ULN in adults (Figure 1). After the vaccinations at the age of 12 months (MenB and MenC), Al levels in plasma and tissues decline slowly and approach baseline (food-only exposure) at about 3 (plasma and liver) and 5 (bone) years of age. As brain is modelled as a sink compartment, the small Al quantities entered into brain persist.

The increase in Al levels due to single (booster) vaccinations in children (at 9 years) and adults (at 19 years) is hardly visible in plasma or tissues (Figure 1). Median ratios (Table 2) indicate a maximum increase over the dietary background exposure of 14% (plasma and liver), 4% (bone) and 3% (brain) in children, and a maximum increase of 4% (plasma), 2% (liver and brain) and 0.4% (bone) in adults.

### 3.2. Al Exposure from Food and Vaccinations Including “Additional HepB at Birth”

The predictions for the scenario that included additional HepB vaccinations is illustrated in Figure 2 (right). The two additional vaccinations at birth and 1 month lead to a moderate (flat) early rise in plasma and liver Al levels, again less pronounced in bone and negligible in brain. The peak in plasma at 2 months of age is therefore elevated and is now the highest at 1.9 (p95: 4.2) µg/L (Cmax), compared to Cmax of 1.6 (3.4) µg/L at 4 months without additional HepB vaccinations (see Figure 2, middle, and Table 2). In all tissues, the concentrations remain still below the initial levels at birth.

### 3.3. Body Burden of Al from Food and Vaccinations After 50 Years

The model-predicted median total amount of Al present in the body of a reference female adult at the age of 50 after continuous dietary intake according to the food-only scenario described in Section 2.1.1. is 5.105 (p5–p95: 0.854–39.280) mg compared to 5.122 (0.862–39.346) mg for the food + vacc scenario (including “additional HepB at birth”, as described in Section 2.1.3). This results in a median increase in body burden due to vaccinations after 50 years of life of 17 µg (0.3%).

## 4. Discussion

These are the first physiologically-based model predictions of concentration–time profiles of Al in plasma and tissues after administration of adjuvanted vaccines in humans on top of an average age-dependent dietary exposure. It is important to emphasise that our predictions are based on a wide range of clinical and physiological data that were used to build and validate the model. All assumptions regarding initial Al levels at birth, physiological growth, Al absorption, and Al bone kinetics following the human age-dependent Ca turnover have been thoroughly validated [4]. In particular, proof has been shown of the model’s qualification for making credible predictions for i.m. administration of Al adjuvanted vaccines in animals [4,25]. Though only sparse data were available for validation of the model performance in infants, the model was considered appropriate for predictions in healthy full-term neonates and infants, as it accounts for the time-based changing physiology with regard to body size, organ volumes and blood flows, including the ontogeny of physiological processes most relevant for Al kinetics, i.e., the maturation of the renal function (GFR) and the age-specific Ca kinetics in bone from birth to adulthood, and for the natal body burden resulting from maternal exposure. Of note is that, due to the model physiology representing the growth of full-term infants (median body weight at birth: 3.5 kg), predictions of Al toxicokinetics in preterm infants are beyond the capabilities of this study.

The predictions demonstrated that single vaccinations in children and adults lead to substantial increases of Al concentration neither in blood nor tissues compared to the dietary background level (maximum increase by 14%).

Vaccinations of healthy full-term infants during the first year of life do moderately increase the overall Al exposure. The maximum rise relative to the dietary intake in infants occurs early (at about 4 months of age) showing a 2-to-3-fold increase in Al levels in plasma, liver and bone. However, this increase is transient and occurs in a period when blood and tissue levels decline (from maternal levels) due to the low intake from infant food and dilution by body growth. It was observed early on that Al levels measured in plasma and bone at birth are not different from those known from adults [26]. For example, Cmax predicted in plasma in vaccinated infants at 4 months (m/p95: 1.6/3.4 µg/L; Table 2) is still within the range of levels measured soon after birth (2–15 µg/L, [4]) or in normal adults (1–10 µg/L; [9]). It is far below Al plasma levels associated with clinical signs of osteomalacia and cholestasis reported from preterm infants on TPN (>20–30 µg/L; [26,27,28]). In particular, this is underpinned by the maximum tissue levels predicted in the important target organs bone and liver that also remain below the ULN of adults and far below critical levels described in the literature. Most importantly, the relative increase in Al concentration in brain by 4% due to the vaccinations is very small. Thus, the main finding of our analysis is that the maximum Al levels predicted in plasma and tissues due to vaccinations in full-term infants during the first year of life do not exceed the levels observed in normal infants soon after birth.

The vaccination schedules in place in the EEA vary [14]. We used the most recent German schedule [13]. The vaccination scheme that includes DTaP (“2 plus 1”) is similar to those of the majority of member states, and represents an early start at 2 months of life. In addition, Al-adsorbed MenC and MenB vaccinations are generally recommended in the first two years of life. Thus, Al exposure from the chosen schedule during the first two years (4.8 mg) is considered representative of other countries. For example, a maximum of 4.5 mg cumulative exposure after 2 years was administered in the recent Danish cohort study [7], and for the US, Mitkus et al. [29] calculated 4.2 mg Al based on the US vaccination schedule in 2011.

In some EU countries hepatitis B vaccination at birth is generally recommended. Our simulation revealed that additional HepB vaccinations at birth only marginally increase plasma and tissue levels, which still remain below the initial levels encountered at birth. The flat rise of plasma Al is mainly attributed to the low dose (0.25 mg Al) and the slow absorption rate. The rise would have been even flatter in cases of use of a product with AH instead of the more quickly absorbed AAHS.

**Figure A1:**
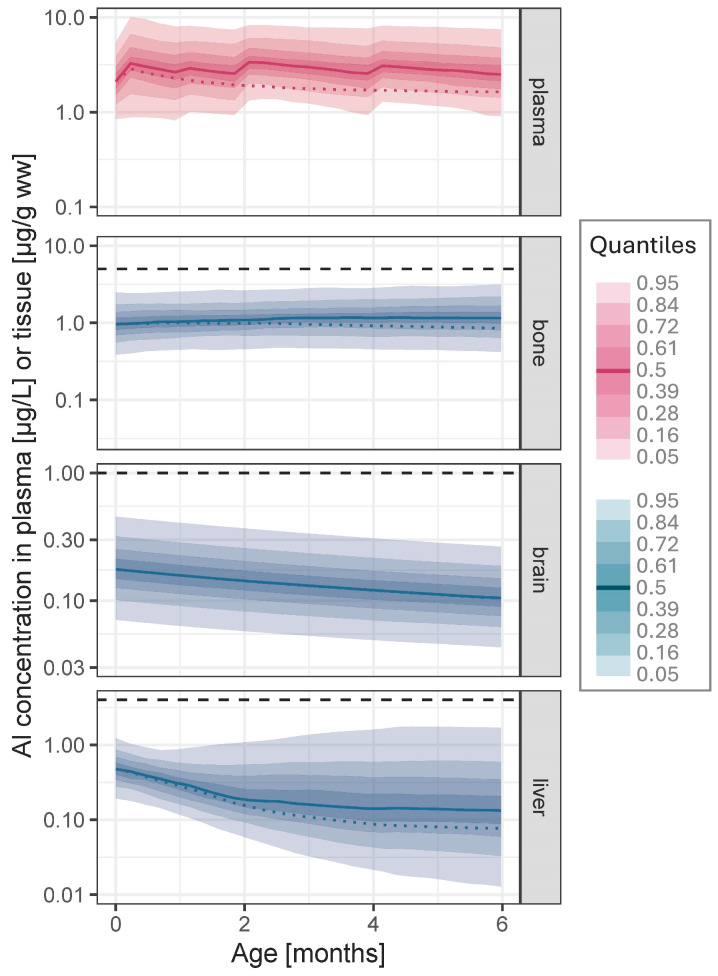
Predicted time courses of Al concentration in plasma, bone, brain and liver following joint exposure from vaccinations (according to the generally recommended vaccination schedule in Germany 2025 [12] including two additional HepB vaccinations after birth (adjuvant type: AAHS); see Table 1 and Figure 2 in the main text) and continuous background dietary intake (food + vacc) by using a maximum dietary exposure of 2 mg/kg/w from birth to the age of 6 months (solid line: Median; coloured shaded areas: Quantiles; horizontal dashed line: Upper limit of normal (see Section 2.3); dotted line: Median time course of “food-only” exposure).

We are aware that the underlying dietary intake determines the total Al levels reached after vaccinations, and the predicted decline in Al concentrations after birth is mainly attributed to the low infant, compared to maternal, food intake assumed. The data we used for the continuous dietary Al exposure over age reflect a rational, average European scenario proven to be in line with reference Al tissue levels in the literature [4]. However, especially for infants (0–6 months of age), our intake assumption (0.1–0.2 mg/kg/w) is not strictly conservative, being a compromise between levels estimated for breast-fed (0.04 mg/kg/w, [12]) and formula-fed average-consuming infants (0.21–0.32 mg/kg/w; [8]). The upper bound estimate for high consumers of infant formulas is reported as 0.52 mg/kg/w [8]), and in cases of soy-based or hypoallergenic formulas, intake could be as high as 2 mg/kg/w [8,12]. In order to cover worst-case assumptions, we investigated the impact of a “maximum infant formula exposure” on our predictions (Figure A1, Appendix A). This scenario would also cover the case of lower intake but potentially much higher oral Al bioavailability in infants as compared to adults. As expected, the high 2 mg/kg/w exposure from infant formulas between 0–6 months of life leads to an elevation of Al levels in both curves; however, the maximum total Al levels in plasma and tissues from food and vaccinations are still within the range of initial levels at birth (e.g., p95 in plasma remains <10 µg/L) and far below levels of concern (>20–30 µg/L; see above). Thus, even in relation to very high and not-declining initial levels, the absolute increases predicted (e.g., 1.1 µg/L in plasma) are still considered moderate.

With respect to the first months of life, our predictions for the Al brain uptake could be challenged by the general scientific claim of incomplete blood–brain barrier (BBB) integrity in early infancy. The BBB represents a multicellular and highly selective permeability barrier system that guarantees brain homeostasis by regulating active transport of nutrients essential for the development of the CNS and provides a defense line against the passage of potentially harmful xenobiotic substances [30,31]. It is present in the majority of brain capillaries and starts forming early during embryogenesis [30]. While the physical barrier of tight junctions can be considered mature at birth, the functional activity of various transporters is modulated during the postnatal period [31]. Thus, since the BBB is the primary route of brain uptake of metals [32] and Al uptake is suspected to be transporter-mediated (see below), Al transport into brain might be altered during postnatal development.

Al^3+^ ions circulating in plasma are mainly (approx. 90%) bound to the transport protein transferrin (Tf) and secondarily (approx. 10%) to citrate [33]. The presence of transferrin receptors on brain capillary endothelial cells (BCECs) provides a means by which plasma transferrin-bound iron crosses the BBB [34]. Transferrin receptor (TfR)-mediated transcytosis is also considered a main transfer route of Al^3+^ ions into brain. A second route is a transporter-mediated uptake of Al^3+^ ions bound to citrate (e.g., by the monocarboxylic acid transporter (MCT-1); [32,35]). These transporters might be affected by BBB maturation: For instance, matching the high iron demand during rapid brain growth, TfR expression and iron uptake was found to be 8 times higher in developing (postnatal day 15) than in adult rats (day 70) [34,36]. Vanucci and Simpson [37] found that MCT-1 protein expression in rats peaks during suckling and declines by 50% during maturation, indicating a doubling of the brain uptake rate of Al citrate during the first weeks.

Both findings may suggest that, due to BBB immaturity also in human newborns, Al transport into brain could be faster than the rate assumed in our model. However, there are data indicating that there is also efflux of Al citrate through the BBB [32], which would mitigate this effect. In addition, data in juvenile rats are in general considered not predictive, since the rat brain is neurologically very immature at birth compared with humans [31]. Of note is that the brain concentration of 0.3 µg/g ww measured in a child after 5 weeks of TPN in the study of Moreno [38] could be well predicted by our model with the “mature BBB” brain intrusion (uptake) rate [4]. This does not support a significantly elevated Al brain uptake rate during the first weeks of life.

**Figure A2:**
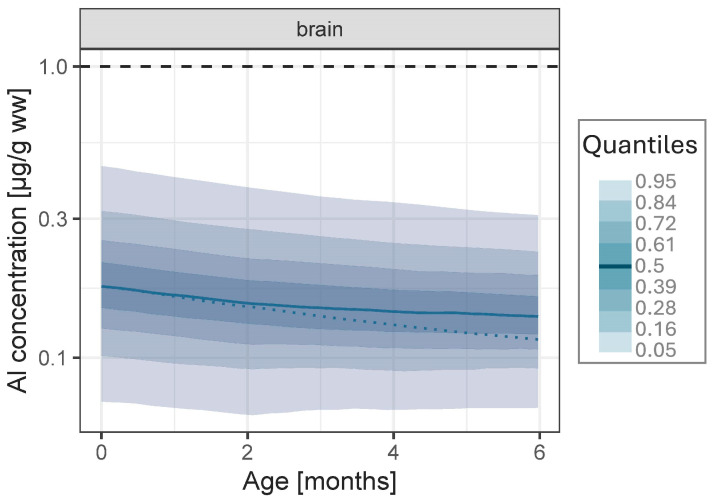
Predicted time courses of Al concentration in brain following joint exposure from vaccinations (according to the generally recommended vaccination schedule in Germany 2025 [12] including two additional HepB vaccinations after birth (adjuvant type: AAHS); see Table 1 and Figure 2 in the main text) and continuous background dietary intake (food + vacc) from birth to the age of 6 months by using a 20-times-faster intrusion rate from blood into brain while maintaining the sink situation (rate outwards remained at zero) (solid line: Median; coloured shaded areas: Quantiles; horizontal dashed line: Upper limit of normal (see Section 2.3); dotted line: Median time course of “food-only” exposure).

Nevertheless, we investigated the theoretical impact of a unidirectional constant increase in BBB transport of Al during the first six months of life by repeating the simulation of the “food and vaccinations including HepB at birth”-scenario (see Section 2.1.3) using a 20-times-faster intrusion rate for Al from blood into brain while conservatively maintaining the sink situation (rate outwards remained at zero). The resulting Al concentrations in brain would be higher in both scenarios (food only: median (p95): 0.116 (0.296) µg/g ww; food + vacc: 0.139 (0.309) µg/g ww; Figure A2, Appendix A). The maximum increase after 6 months due to vaccinations would be 1.2-fold (median difference: 0.024 µg/g ww). The total level would still not exceed the initial level range of infants soon after birth.

Another concern expressed frequently is the contribution of Al from vaccinations to the “body burden” over a lifetime (as a possible cause for neurodegenerative diseases). According to the PBTK model, the brain is the only organ where total retention can occur, whereas accumulation in bone is transient. Due to the continuous age-dependent process of bone remodelling, Al (following Ca) is assumed to be slowly but steadily released from bone. The specific bone submodel implemented in the PBTK model reflects these kinetic processes [4,15]. Our calculated body burden of 5 mg at the age of 50 (i.e., the amount of Al present in the body at that time) is perfectly in line with Priest et al. [39] who estimated an accumulation of 2–7 mg from oral dietary exposure during adult life based on their 26Al-retention studies. Of note is that this body burden does not include Al particles, e.g., those present in lungs or lymph nodes deriving from inhalative exposure of Al dust, which is, however, included in higher burden estimates based on extrapolations from chemical analysis of all tissues (35–40 mg; [40]). Most importantly, our predictions clearly illustrate that the contribution of vaccinations recommended for routine immunisation from birth to adulthood to the body burden is negligible.

The latest attempt to predict and assess the risk of Al exposure in infants from vaccinations was done by Mitkus et al. in 2011 [29]. They estimated the total body burden of aluminium retained from dietary and vaccine exposures and compared it to that calculated from the minimal risk levels (MRLs) established by the US Agency for Toxic Substances and Disease Registry (ATSDR) derived from animal studies.

Similarly to our assumptions, they accounted for a background dietary intake and a baseline level of Al in the blood of newborns. In contrast, we additionally based the initial levels at birth also on measurements in tissues other than blood [4]. To estimate the i.m. absorption rate, Mitkus et al. used data from just two rabbits, whereas our estimates were based on additional adjuvant-specific injection site measurements in rats and monkeys [4].

The main advance of our approach, however, is that it allows for a prediction of Al concentrations over time at the tissue level in relevant target organs like bone, the brain, and the liver, in contrast to their calculations of total Al amounts in the whole body based on an empirical retention function. Furthermore, the physiologically-based approach enables reliable predictions for children and even neonates. Physiological changes with age related to childhood growth and maturation processes (GFR and calcium-oriented Al bone metabolism) are dynamically accounted for.

Through the use of a toxicokinetic state-of-the-art method, our findings corroborate the previous conclusion drawn by Mitkus et al. [29] that episodic exposures to Al-containing vaccines continue to be extremely low-risk to infants.

A recent Danish population-based cohort study provides complementary epidemiological support for this conclusion: Andersson et al. [7] did not find evidence that cumulative Al exposure from vaccination during the first 2 years of life was associated with an increased risk (at least a moderate-to-strong increase in risk (>30%)) for any of 50 autoimmune, atopic, and neurodevelopmental disorders assessed (including asthma, atopic dermatitis, rheumatoid arthritis, autism spectrum disorder, and attention deficit–hyperactivity disorder).

Our model-based predictions of maximum Al tissue level increases due to vaccination, and these epidemiological results complement each other very well. Our finding of very small brain Al increases supports the negative findings by Andersson et al. regarding CNS outcome. In addition, we provide supportive evidence that toxicologically relevant Al levels are also not to be expected in bone or liver, the toxicological impacts of which were not investigated in Andersson’s study.

## 5. Conclusions

In summary, Al concentrations predicted in plasma and tissues after joint exposure of Al from recommended vaccinations with Al-adjuvanted vaccines and continuous dietary background intake from (full-term) birth to adulthood remain far below levels which might be associated with toxicity. Even the moderate increase in Al exposure due to vaccinations during the first two years of life is considered safe, since maximum Al levels predicted in plasma and tissues did not exceed normal levels measured in infants soon after birth or known from adults. Thus, predicted Al increases are in no way commensurate to the life-saving benefit of the vaccinations. We conclude that, even though the cumulative Al amount administered through vaccination has increased over the years, regarding Al toxicokinetics, the current recommended vaccination schedules can be considered safe.

The model used in this study has proven a valuable tool for simulations of Al exposure after vaccinations or subcutaneous immune therapy (SCIT; [11]) and may further support risk assessment by regulatory bodies, especially with respect to changing/expanding vaccination and treatment schedules.

## Figures and Tables

**Table 1:** Al exposure from vaccinations from birth to 50 years of age used in the simulation scenarios based on the vaccination schedule 2025 [13] including additional HepB vaccinations at birth (italic font) and the highest Al amount of available products in Germany; Al adjuvant type (AH: aluminium hydroxide; AP: aluminium phosphate; AAHS: amorphous aluminium hydroxyphosphate sulfate) and Al amount per vaccine dose were retrieved from the Summaries of Product Information.

Age		Vaccination	Al Amount (mg) per Vaccine Dose			Total Al Dose (mg)
Month	Year		AH	AP	AAHS	
*0*		*HepB*			*0.25*	*0.25*
*1*	*0.08*	*HepB*			*0.25*	*0.25*
2	0.2	DTaP-IPV-Hib-HepB	0.5	0.32		0.82
2	0.2	PCV13/15		0.125		0.125
2	0.2	MenB	0.5			0.5
4	0.3	DTaP-IPV-Hib-HepB	0.5	0.32		0.82
4	0.3	PCV13/15		0.125		0.125
4	0.3	MenB	0.5			0.5
11	0.9	DTaP-IPV-Hib-HepB	0.5	0.32		0.82
11	0.9	PCV13/15		0.125		0.125
12	1.0	MenB	0.5			0.5
12	1.0	MenC	0.5			0.5
60	5	TdaP	0.3	0.2		0.5
108	9	HPV (1st)			0.5	0.5
108	9	TdaP-IPV	0.3	0.2		0.5
120	10	HPV (2nd)			0.5	0.5
228	19	TdaP	0.3	0.2		0.5
348	29	Td	0.5			0.5
468	39	Td	0.5			0.5
588	49	Td	0.5			0.5
		**Total**	**5.9**	**1.9**	**1.0 (** ***1.5*** **)**	**8.8 (** ***9.3*** **)**

**Table 2:** Al concentrations predicted in plasma, bone, brain and liver following joint exposure from vaccinations (according to the generally recommended vaccination schedule in Germany 2025 [13]; see Table 1) and continuous background dietary intake (“food + vacc”; see Figure 1 and excerpt in Figure 2, middle) compared to dietary intake only (“food only”) at the timepoint of the highest median difference between both curves stratified into three age periods (m: median; p95: 95% quantile; m diff: difference of medians (m (food + vacc) − m (food only)); m ratio: ratio of medians (m (food + vacc)/m (food only)); ww: wet weight).

Age Period (Years)	Al Exposure	Al Concentration											
		Plasma (µg/L)			Bone (µg/g ww)			Brain (µg/g ww)			Liver (µg/g ww)		
		m	p95	m Diff (m Ratio)	m	p95	m Diff (m Ratio)	m	p95	m Diff (m Ratio)	m	p95	m Diff (m Ratio)
Infant (0–2)	food + vacc	1.618	3.433	1.140 (3.39)	0.668	1.579	0.246 (1.58)	0.073	0.179	0.003 (1.04)	0.066	0.713	0.041 (2.64)
	food only	0.478	1.501		0.422	1.146		0.070	0.176		0.025	0.266	
Child (9–10)	food + vacc	1.006	3.733	0.121 (1.14)	0.411	2.944	0.016 (1.04)	0.096	0.227	0.003 (1.03)	0.049	0.934	0.006 (1.14)
	food only	0.885	3.677		0.395	2.860		0.093	0.226		0.043	0.902	
Adult (19–20)	food + vacc	1.380	5.961	0.049 (1.04)	0.533	4.245	0.002 (1.004)	0.136	0.373	0.003 (1.02)	0.068	1.318	0.001 (1.02)
	food only	1.331	5.936		0.531	4.236		0.133	0.366		0.067	1.308	

## Data Availability

The original data presented in the study are openly available in a publicly available repository [https://doi.org/10.5281/zenodo.18442705].

## Abbreviations

The following abbreviations are used in this manuscript:

AH	Aluminium hydroxide
AP	Aluminium phosphate
AAHS	Amorphous aluminium hydroxyphosphate sulfate
DTaP-IPV-Hib-HepB	Diphtheria, tetanus, acellular pertussis, inactivated poliovirus, Haemophilus influenzae type b, and hepatitis B combination vaccine (6-in-1)
HepB	Hepatitis B vaccine
HPV	Human papillomavirus vaccine
Td	Tetanus & diphtheria vaccine, adult/adolescent formulation
Tdap	Tetanus, diphtheria and acellular pertussis vaccine, adult/adolescent formulation
Tdap-IPV	Diphtheria, tetanus, acellular pertussis and inactivated poliovirus combination vaccine, adult/adolescent formulation
MenB	Serogroup B meningococcal vaccine
MenC	Serogroup C meningococcal vaccine
PCV13/15	Pneumococcal Conjugate Vaccine (13-/15-valent)
